# Home-based arm cycling exercise improves trunk control in persons with incomplete spinal cord injury: an observational study

**DOI:** 10.1038/s41598-023-49053-w

**Published:** 2023-12-13

**Authors:** Joeri F. L. van Helden, Emma Alexander, Hélio V. Cabral, Paul H. Strutton, Eduardo Martinez-Valdes, Deborah Falla, Joy Roy Chowdhury, Shin-Yi Chiou

**Affiliations:** 1 Centre of Precision Rehabilitation for Spinal Pain (CPR Spine), School of Sport, Exercise and Rehabilitation Sciences, College of Life and Environmental Sciences, University of Birmingham, Edgbaston, Birmingham, B15 2TT UK; 2 The Royal London Hospital, Barts Health NHS Trust, London, UK; 3 Department of Clinical and Experimental Sciences, Università degli Studi di Brescia, Brescia, Italy; 4 Department of Surgery & Cancer, Faculty of Medicine, Imperial College London, London, UK; 5 Midland Centre for Spinal Injuries, The Robert Jones and Agnes Hunt Orthopaedic Hospital NHSFT, Oswestry, UK

**Keywords:** Neurophysiology, Trauma

## Abstract

Arm cycling is used for cardiorespiratory rehabilitation but its therapeutic effects on the neural control of the trunk after spinal cord injury (SCI) remain unclear. We investigated the effects of single session of arm cycling on corticospinal excitability, and the feasibility of home-based arm cycling exercise training on volitional control of the erector spinae (ES) in individuals with incomplete SCI. Using transcranial magnetic stimulation, we assessed motor evoked potentials (MEPs) in the ES before and after 30 min of arm cycling in 15 individuals with SCI and 15 able-bodied controls (Experiment 1). Both groups showed increased ES MEP size after the arm cycling. The participants with SCI subsequently underwent a 6-week home-based arm cycling exercise training (Experiment 2). MEP amplitudes and activity of the ES, and movements of the trunk during reaching, self-initiated rapid shoulder flexion, and predicted external perturbation tasks were measured. After the training, individuals with SCI reached further and improved trajectory of the trunk during the rapid shoulder flexion task, accompanied by increased ES activity and MEP amplitudes. Exercise adherence was excellent. We demonstrate preserved corticospinal drive after a single arm cycling session and the effects of home-based arm cycling exercise training on trunk function in individuals with SCI.

## Introduction

A significant proportion of people with spinal cord injury (SCI) exhibit reduced control of their trunk muscles^1,2^. This can severely disrupt upper limb^3,4^ and locomotor function since the ability to maintain upright trunk stability is essential for carrying out functional and recreational activities^5–7^ such as feeding, dressing, transferring, and playing sports. However, standard interventions for trunk rehabilitation are complex and require considerable input from experienced therapists and care-partners to assist the patient in performing the specific exercises^8,9^. These requirements limit the time in which the rehabilitation can be performed, impeding recovery. Hence, there is a need to identify new approaches for trunk rehabilitation after SCI.

It is well established that there are functional interactions between the trunk and upper limbs^10,11^. For example, trunk muscles are activated prior to or concurrent with arm movements to minimise postural displacement^12,13^ and to assist movements of the arms^14,15^. Additionally, activation of trunk flexors and extensors was reported during upper-body exercises, such as arm cycling, and boxing and battle rope exercise, in individuals with SCI^16,17^. Several lines of evidence suggest physiological interactions between muscles of the trunk and upper limbs; using transcranial magnetic stimulation (TMS) over the primary motor cortex, studies reported increased motor evoked potential (MEP) amplitudes in the trunk extensors and flexors during voluntary contractions of the muscles of the upper limbs in healthy adults and in people with chronic incomplete SCI^18–21^. Moreover, prior work demonstrated an increase in MEP amplitudes in thoracic erector spinae (ES) after cessation of 30 min of unilateral rhythmic arm cycling in healthy adults and this facilitatory effect lasted for 20 min post-cycling^22^. These results suggest acute neuroplasticity in the corticospinal pathways projecting to the trunk muscles induced by voluntary contractions of the arm muscles. Inducing neuroplasticity in the corticospinal tract has been shown to promote voluntary movement and functional recovery^23–25^. For example, strengthening transmission in the neural pathways to the injured muscles, improving voluntary motor output, resulting in enhanced function in people with SCI and stroke^25,26^. Hence, we hypothesised that exercise involving arm movements will strengthen the corticospinal pathways to the trunk muscles, leading to improved trunk motor control in people with SCI.

Arm cycling exercise is a form of volitional exercise commonly used by individuals with SCI for cardiorespiratory fitness^27^. The exercise is simple and can be performed outside a clinical setting by individuals without supervision from professionals, positing an opportunity to overcome the barriers of current approaches for trunk rehabilitation. Hence, the first aim of this study was to compare the effects of single session of arm cycling on corticospinal excitability of the ES musles in individuals with chronic incomplete SCI and able-bodied controls. We hypothesised that MEP amplitudes of the ES would increase after single session of arm cycling in both individuals with SCI and able-bodied controls. The second aim of this study was to examine the effects of a 6-week home-based, unsupervised arm cycling exercise training on corticospinal excitability and neuromuscular function of the ES in individuals with chronic incomplete SCI. We hypothesised that corticospinal drive to the ES will increase and the volitional control of the trunk muscles will improve during functional tasks after the training.

## Methods

The study protocol was approved by West Midlands–Edgbaston Research Ethics Committee (19/NI/0075) and performed in accordance with the Declaration of Helsinki. Written informed consent was obtained from all participants prior to data collection.

### Participants

Fifteen adults with a stable incomplete cervical or thoracic SCI (see results for demographics) were recruited from the Midland Centre for Spinal Injuries and from social media between 2019 and 2022 to participate in both Experiments 1 and 2, based on a power calculation drawn from a previous study^22^ suggesting a sample size of 15 was sufficient to detect a pre-post effect for peak-to-peak amplitudes of MEPs in the thoracic ES with 90% power and a 5% level of significance. Additionally, fifteen healthy adults (21.1 ± 0.5 years old, with 7 males) with no history of neurological conditions or disorders were recruited for experiment 1 as controls. Inclusion criteria for participants with SCI were: (1) categorised by the American Spinal Cord Injury Impairment Scale (AIS) as AIS C or D (motor incomplete injury), (2) > 12 months from the time of injury, (3) having sufficient motor function of upper extremities to voluntarily move the pedals of the arm bike to perform arm cycling exercise, and (4) able to maintain upright seated posture with back supported and without using the arms for balance for > 10 s. Participants were excluded if they had contraindications to TMS (i.e., previous brain injury or brain surgery, metal implants or medical devices placed inside the head, history of epilepsy or seizure, actively taking antidepressants or other neuromodulatory drugs, or pregnancy)^28^. All assessments were performed in the laboratories at the University of Birmingham.

### Bipolar surface electromyography (EMG)

Muscle activity was recorded bilaterally from the ES at the 12th thoracic vertebral level (T12) and biceps brachii using surface electromyography (Delsys^®^ Bagnoli-2 EMG system). The skin was cleaned with alcohol wipes (GAMA Healthcare, Hertfordshire, UK). Two single differential surface EMG sensors (DE-2.1, Delsys Inc., USA) with a contact surface of 10 mm × 1 mm and an inter-electrode distance of 10 mm were placed over the belly of the biceps brachii, and over the ES at two centimeters lateral to the T12 spinous process. A reference electrode was placed on the left iliac crest. EMG signals were amplified by a factor of 1000, bandpass filtered at 20–450 Hz, and sampled at 1 kHz using a micro 1401 data acquisition unit (Cambridge Electronica Design, Cambridge, UK). The data were acquired, stored, and analysed by Signal v6.05 or Spike v10 software.

### Transcranial magnetic stimulation (TMS)

TMS pulses were delivered via a Magstim 200^2^ monophasic stimulator (The Magstim Company Ltd., Whitland, UK) through a double-cone coil (loop diameter: 110 mm), handle pointing backward and ~ 45° away from the midline to induce a current flow in the anteromedial direction. The coil was placed over the primary motor cortex ipsilateral to the less affected arm (participants with SCI) or the dominant arm (controls) to elicit MEPs in the contralateral ES. To determine the less affected arm in the participants with SCI, we tested strength of the biceps brachii and triceps brachii from both arms with manual muscle testing and the arm graded with a higher strength was determined as the less affected arm. The optimal position (hot spot) for the coil was determined as the point where the largest MEP was elicited in the contralateral ES. This coil position was saved in a neuro-navigation system (Brainsight 2, Rogue Research Inc, Montreal, Canada) and used throughout the experiment and at post-assessment for consistency of the coil placement. TMS intensity was determined to evoke MEPs in the ES at peak-to-peak amplitudes of ~ 0.1 mV. In some participants with SCI whose ES MEPs were less than 0.1 mV, an intensity of 100% maximal stimulator output was used.

### Experimental procedures

#### Experiment 1. Modulation of corticospinal excitability of the ES following a single session of arm cycling

**Figure 1:**
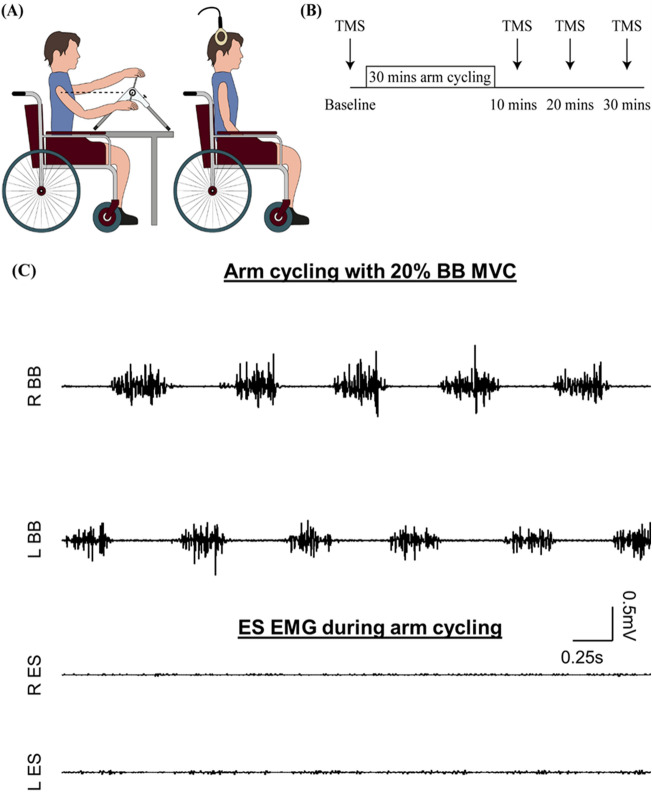
Experimental setup of Experiment 1. (**A**) Schematic illustration of the setup of arm cycling (left) and TMS stimulation (right). (**B**) A study diagram showing the time course of experiment 1. (**C**) Raw EMG traces recorded from bilateral erector spinae (ES) and biceps brachii during arm cycling with ~ 20% of maximal voluntary contraction of the biceps brachii.

An arm ergometer (Pedal Exerciser with Digital Display, NRS Healthcare, Coalville, UK) placed on a height-adjustable table was used for arm cycling. The height and distance of the ergometer were adjusted individually so that the arm crank shaft was at the same height as the shoulders and the maximal pedal distance was with the elbow softly bended to minimise movements of the scapula, shoulder and upper body. Participants performed 2–3 brief (~ 2 s) maximal voluntary contractions of the elbow flexors, with the shoulder at neutral position and the elbow flexed at 90° prior to the exercise. EMG amplitudes obtained from the maximal voluntary contractions were calculated as the root mean square (RMS) amplitude in a 500 ms window centreed at the peak amplitude. All participants underwent 30 min of arm cycling exercise at 60 revolutions per minute (RPM) in seated position without back support in our laboratory (Fig. 1A). Breaks were given when needed. The resistance of the arm bike was set to require activity of the biceps brachii EMG in the pulling back phase (flexion phase) of the arm crank movement at ~ 20% of the maximal voluntary contraction of the less affected arm for the participants with SCI or the dominant arm for the controls (Fig. 1C)^20,21^. To examine the effect of the arm cycling on corticospinal excitability of the ES, TMS pulses were delivered before, and 10, 20, and 30 min after the arm cycling (Fig. 1B) when participants were seated and relaxed (with the back supported) in the chair. Ten TMS pulses were delivered at 4 s intervals at each time point.

*Data analysis.* Peak-to-peak amplitudes of MEPs from the ES were averaged and measured for each time point and expressed as a percentage of the baseline MEP amplitude of the ES (before the arm cycling). Pre-stimulus baseline EMG obtained from ES was calculated as RMS amplitudes in 100 ms window before TMS to ensure that ES motoneuronal excitability was the same across all time points. Latencies of ES MEPs were determined as the point where rectified EMG traces exceeded 2 SD of the mean pre-stimulus baseline EMG.

#### Experiment 2. The effects of arm cycling exercise training on trunk motor control and corticospinal excitability of the ES in participants with SCI

Participants with SCI who completed Experiment 1 were recruited into Experiment 2 to undertake a home-based arm cycling exercise training consisting of 5 × 30 min of arm cycling at 60 RPM in a week for 6 weeks. The initial resistance of the arm cycling was at ~ 20% of the maximal voluntary contraction of the biceps brachii, measured in Experiment 1. Participants were instructed to increase the resistance of the arm bike progressively in order to maintain the exercise intensity at a rating of perceived exertion of 4 based on the modified CR-10 Borg Scale^29^ throughout 6 weeks of training period. Participants were given an exercise diary to document their exercise adherence. They were contacted by the research team (JFLvH, EA) every 1–2 weeks to ensure there were no issues with the exercise. Participants were assessed before (pre-) and after (post-) the training in the laboratories at University of Birmingham.

##### Assessments

**Figure 2:**
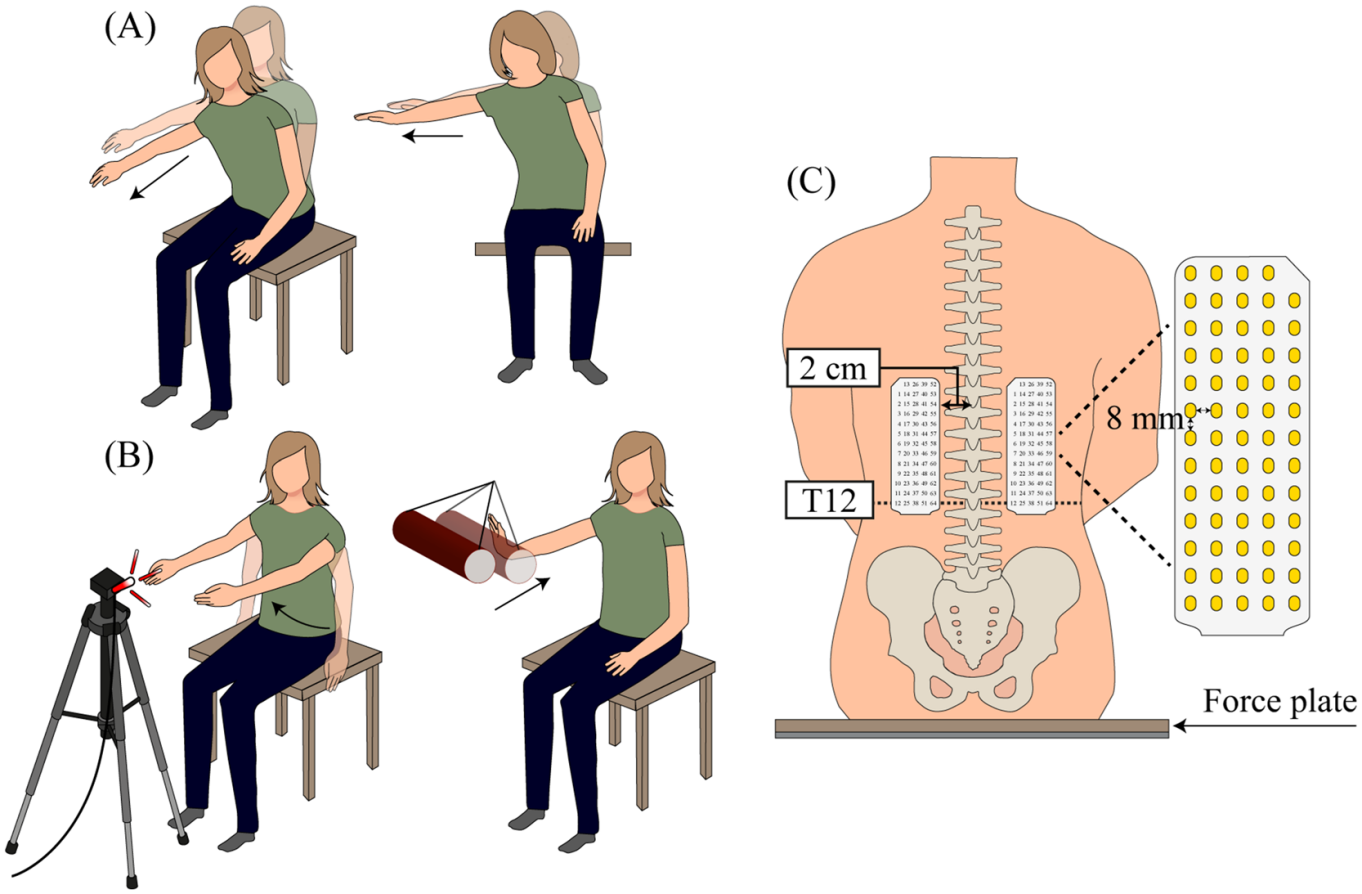
Functional assessment and HDEMG placement. (**A**) Multidirectional reaching tasks in the forward (left) and lateral (right) directions. (**B**) Perturbation tasks: rapid shoulder flexion task (left) and external perturbation task (right). For the rapid shoulder flexion task, illumination of a light-emitting diode signaled to the participant to flex the shoulders to approximately 90° as fast as possible. For the external perturbation task, a cylinder with weights was pulled back and released and swayed into the participant’s extended hand. (**C**) Bilateral placement of HDEMG electrode grids in a vertical orientation, aligned with the ES (T12 level and upward).

Participants underwent neurophysiological and functional assessments. For the neurophysiological assessment, participants received single TMS pulses eliciting MEPs in the ES at peak-to-peak amplitudes of ~ 0.1 mV while they were seated and relaxed in a chair. If a peak-to-peak amplitude of 0.1 mV was unable to be achieved, an intensity of 100% maximal stimulator output was used. The stimulus intensity was the same at pre- and post-assessment. The coil position stored in the navigation system at pre-assessment was used at post-assessment so that the coil placement was the same between sessions. For the functional assessment, participants performed multidirectional reaching tasks and perturbation tasks while seated on a custom-made chair embedded with a force plate and reflective markers attached bilaterally over the ulnar styloid processes and over the first thoracic spinous process^30^. Their torso was unsupported, and feet were placed flat on the floor or a step, with hips and knees flexed at 90°. Participants were asked to reach forward (Fig. 2A left) and to the side (Fig. 2A right) with the less affected arm as far as they could without losing balance for 3 times^30^. In the first perturbation task, participants raised their arms as fast as possible in response to a light-emitting diode five times (rapid shoulder flexion; Fig. 2B left). In the external perturbation task, a pendulum with a weight of ~ 5% of the body mass of the individuals was released from a 45° angle towards the extended arm of the participants five times (Fig. 2B right)^30^. Kinematics of the wrist, trunk, and centre of pressure, and activation of the ES during the tasks were recorded using a 3-D motion capture system and high-density surface electromyography (HDEMG), respectively. HDEMG was chosen over conventional bipolar EMG as it allows recording of muscle activity from a larger portion of the muscle compared to the conventional EMG^31^, providing greater reliability of amplitude estimations^30^, which is ideal for repeated measurements.

##### Kinematics

Trunk and wrist movements during the tasks were collected with a motion capture system (Smart-DX 6000, BTS Bioengineering Corp, Quincy, MA, USA) and seated ground reaction movements were recorded with a force plate (BTS P6000, BTS Bioengineering Corp, Quincy, MA, USA), both operating at 250 Hz. BTS Bioengineering Corp software tools were used to record and export data. A transistor–transistor logic switch was connected for offline data alignment and to indicate trial start/end.

##### HDEMG

Activity of bilateral ES was measured in monopolar mode using an HDEMG amplifier (Quattrocento, OT Bioelettronica, Turin, Italy), sampled at 2048 Hz, bandpass filtered at 10–500 Hz with a 3 dB cutoff frequency, 150-gain applied, and 16-bit A/D converted. Input resistance was > 10^11^Ω, input-referred noise was < 4 µV, and the common mode rejection ratio was > 95 dB. Two 64-channel grids (GR08MM1305, OT Bioelettronica, Turin, Italy), organised in 13 columns by 5 rows with an 8 mm interelectrode distance and 1 mm diameter were placed on bilateral ES, leaving a 2 cm space between the grid and the 12th thoracic spinous process, with the top of the grid extending to approximately the 8th thoracic spinous process (Fig. 2C). Grid and skin preparation followed previously reported procedures, starting with attaching an adhesive foam matrix to the electrode grid^30^. The circular cavities in the foam matrix were then filled up with conductive paste (AC Cream, Spes Medica, Genoa, Italy) using a plastic card. In case it was necessary, the skin of the participant was shaved prior to the application of an abrasive skin cleaner (Nuprep Skin Prep Gel, Weaver and Company, CO, USA) and alcohol-based skin wipes (GAMA Healthcare, Hertfordshire, UK). Distance from bodily landmarks such as birthmarks or spinous processes to the electrode grids were measured to ensure consistent placement of the electrode grids in the post-assessment. Ground electrodes (Ambu WhiteSensor WS, Ballerup, Denmark) were placed over bilateral iliac crests and the 7th cervical spinous process. EMG data was recorded with OT Biolab + v1.5.7.

##### Data analysis

The analysis procedure for the ES MEP amplitudes was the same as described in Experiment 1. Kinematic and HDEMG data from the functional assessment were processed in MATLAB R2021a (Mathworks, Natic, MA, USA). EMG signals were filtered (second-order Butterworth filter 20–350 Hz) before visually examining the monopolar EMG channels. Noisy channels were interpolated when possible. As a criterium, at least two non-noisy channels in neighboring rows and one channel in the same column surrounding the noisy channel had to be available to perform the interpolation. If this was not possible, the noisy channel was removed instead in the second visual inspection (see below). Subsequently, the differential of the monopolar channels across rows was calculated, followed by a second visual examination to remove poor-quality channels (3.72% of total number of channels).

Multidirectional reaching tasks were segmented into a reaching phase (baseline position to furthest reaching point) and returning phase (furthest reaching point to baseline position). The analysis window for the rapid shoulder flexion task was based on the onset of the wrist motion marker to the point where the shoulders reached full flexion. The onset was determined as the point at which the wrist motion signal (less affected side) crossed a threshold defined as the baseline values (average of 500 ms before the light-emitting diode stimulus) plus three standard deviations of that baseline^32^.The impact point in the external perturbation task was derived from a clear peak in the participant’s wrist motion data from which two analysis windows were calculated with respect to the impact point: anticipatory postural adjustment window (APA; − 100 to 50 ms) and compensatory postural adjustment window (CPA; 50 to 200 ms)^33^.

RMS amplitudes were calculated for active channels, which were defined as those channels with a RMS amplitude higher than 70% of the maximum RMS amplitude^34^. The average amplitude of these channels was then considered as the global RMS amplitude. To facilitate comparisons of EMG amplitudes between different days (pre vs. post), we normalised the RMS amplitudes of the ES obtained from the tasks to the RMS amplitudes during static sitting (0.5 s window) acquired on the day and expressed as a percentage.

For kinematic measurements, maximal displacement of trunk and wrist markers, as well as the centre of pressure displacement, were calculated from the motion data as a difference between the minimum and maximum values in the anterior/posterior direction (forward reaching, rapid shoulder flexion, and external perturbation task) and medial/lateral direction (lateral reaching) in each repetition. Total trajectory of the markers and centre of pressure were calculated by summing the absolute differences in both the anterior/posterior and medio-lateral directions.

### Statistical analysis

Statistical analyses were conducted in SPSS v29 (IBM Corp., Armonk, NY, USA). Normality assumptions were evaluated with the Shapiro–Wilk test and non-parametric tests were applied if normality was violated. In Experiment 1, the mixed-model repeated measures analysis of variance (ANOVA) was used to examine a main effect of time (before, and 10, 20, and 30 min after arm cycling) and an interaction of time x group (SCI vs. control) on MEP amplitudes of the ES, MEP latencies, and pre-stimulus ES EMG. In Experiment 2, paired-samples *t*-tests or Wilcoxon signed-rank tests were used to compare pre- and post ES MEP amplitudes and latencies, pre-stimulus background ES EMG, maximal displacement of the wrist (reaching distance), trunk and centre of pressure, and the reaction time (rapid shoulder flexion). A paired *t* test was also used to compared normalised RMS amplitudes during the rapid shoulder flexion task at pre- and post-assessment. Furthermore, repeated measures ANOVAs were applied to determine the effect of time (pre vs. post) and phase (reaching and returning phase in multidirectional reaching tasks) or window (APA vs. CPA in the external perturbation task) on the trajectory of the trunk and centre of pressure, and on normalised RMS global amplitudes of the ES. Post-hoc tests with Bonferroni’s correction were applied for pairwise comparisons when needed. Data are presented are presented as mean ± SD or median and range in the text. Effect sizes (η^2^p) are reported, and significance was set at p < 0.05.

## Results

### Overview

The study comprised two experiments. Experiment 1 was a cross-sectional study comparing the modulation of arm cycling exercise on corticospinal excitability of the ES between healthy adults and individuals with chronic, incomplete SCI to assess whether there is neuroplasticity in the corticospinal pathways projecting to the trunk muscles induced by voluntary contractions of the arm muscles in those individuals. Experiment 2 was a single-arm, observational study to examine whether a 6-week arm cycling exercise training leads to improvement in trunk control and changes in the corticospinal excitability of the ES in the individuals with SCI.

#### Experiment 1

Baseline demographic data of the 15 SCI participants are shown in Table 1 (age 55.4 ± 13.6 years; 13 males; 8.2 ± 14.3 years post-injury). Of the 15 SCI individuals, 10 had an AIS C injury (66.67%), and 5 AIS D (33.33%). In addition, 10 SCI individuals had a cervical injury (66.67%; C3–C7), and 5 had a thoracic injury (33.33%; T2-T11). Characteristics of the healthy control group were 21.1 ± 0.5 years old, with 7 males. The control group was significantly younger (Z = − 4.700, *p* < 0.001) and consisted of significantly more females (Z = − 2.285, *p* = 0.022).

**Table 1:** Demographics of participants with SCI.

ID	Sex	Age (years)	AIS score	Level of injury	Time since injury (years)	Experiment 1 or 2
P01	M	53	C	C5-6	4	1,2
P02	F	71	D	C6-7	59	1,2
P03	M	80	D	T4	7	1,2
P04	M	59	C	C5-6	3	1,2
P05	M	23	C	C4-5	4	1
P06	M	47	C	C3-4	5	1,2
P07	M	46	C	C5-7	3	1,2
P08	M	49	C	C3-4	4	1,2
P09	M	52	C	T2	7	1
P10	M	69	D	T7	10	1
P11	F	50	C	T2	2	1,2
P12	M	52	D	C3-4	2	1
P13	M	63	D	C3-7	7	1,2
P14	M	50	C	C4-5	1	1,2
P15	M	67	C	T11	5	1,2

M: male, F: female, AIS: the American Spinal Injury Association Impairment Scale, C: cervical, T: thoracic.

**Figure 3:**
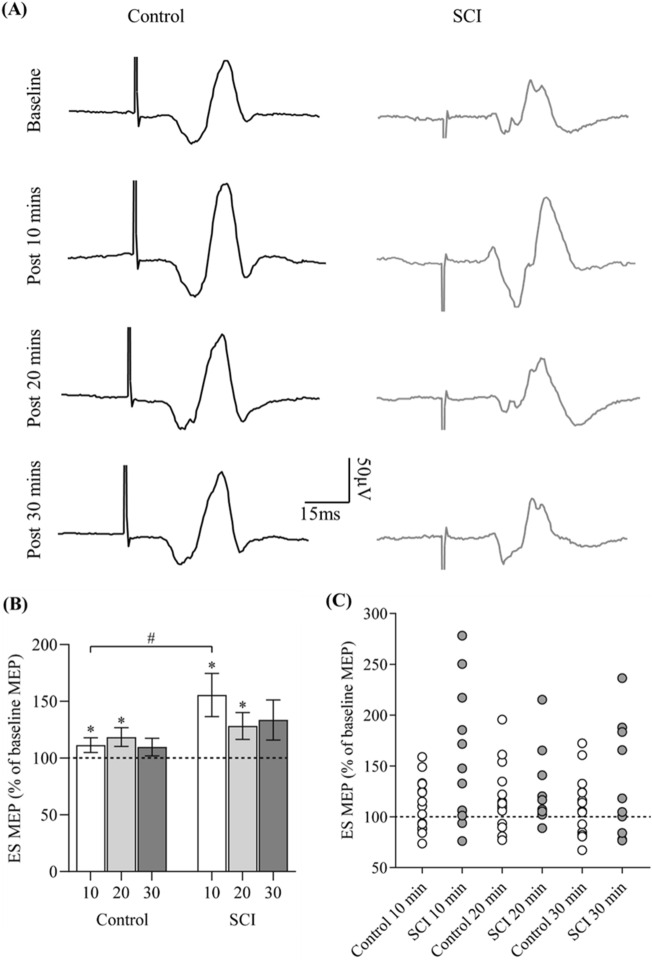
Results of experiment 1. (**A**) Representative MEP traces in the ES obtained from a control participant (black traces) and a participant with SCI (grey traces) at baseline, and at 10-, 20-, and 30-min post arm cycling exercise. (**B**) Group mean results showing ES MEP at 10-min (control [C] = 15; SCI = 12), 20-min (C = 15; SCI = 10), and 30-min (C = 15; SCI = 10) after the arm cycling. (**C**) Individual data demonstrating that most participants with SCI show ES MEP amplitudes above the baseline and the facilitation is decreased along the course of time. The abscissa shows the time point and the horizontal dashed line indicates the ES MEP size at baseline. Error bars represent the standard error of the mean. *p < 0.05, comparisons with baseline; ^#^*p* < 0.05 between groups.

Two participants with SCI (P03, P05) did not have visible ES MEP before or after the arm cycling and their data were not included in the analysis. One participant (P14) did not complete 30 min of arm cycling in the lab, due to lack of appropriate gripping aids available on the day, and the data were not included in the analysis. Figure 3A illustrates raw ES MEP traces from a representative control participant and participant with SCI at baseline, and at 10-, 20-, and 30-min after arm cycling exercise. Note that amplitudes of ES MEPs increase in both participants after the exercise. Group results revealed an effect of time (*F*_3,69_ = 5.25, *p* = 0.003; η^2^p = 0.19) and an interaction of time x group (*F*_3,69_ = 2.79, *p* = 0.03; η^2^p = 0.11) on the MEP amplitudes of the ES, with medium to large effect sizes. Post-hoc tests demonstrated that the MEP amplitudes of the ES were greater at 10- (corrected *p* = 0.003) and 20-min (corrected *p* = 0.009) post exercise compared with the baseline. ES MEP amplitudes were not different between the baseline and 30-min post exercise (corrected *p* = 0.06; Fig. 3B). Additionally, post-hoc results showed that the ES MEP amplitudes were greater at 10-min post exercise in participants with SCI than in the controls (*p* = 0.04; Fig. 3B), while they were not different between the groups at 20- or 30-min post exercise (both *p* > 0.05). Note that most participants with SCI show ES MEPs above the baseline (dotted line) at 10-min after the arm cycling, with the facilitatory effect deteriorating along the course of time (Fig. 3C). Furthermore, there was no effect of time (*F*_3,69_ = 1.05, *p* = 0.38) or an interaction of time x group (*F*_3,69_ = 1.87, *p* = 0.14) on the MEP latencies. Overall, MEP latencies were longer in individuals with SCI (25.60 ± 6.18 ms) than in the controls (14.86 ± 1.54 ms; Z = -3.97; *p* < 0.001). There was no difference in background EMG in ES across all time points (*F*_3,75_ = 0.94, *p* = 0.43).

#### Experiment 2

Of 15 participants with SCI participating in the experiment 2, four participants did not complete the training: two were lost from the study due to the COVID-19 national lockdown, one participant did not have enough time to commit to the intervention, and one participant discontinued due to pre-existing shoulder instability. For the remaining 11 participants (age 57.7 ± 11.2 years; 9 males; 8 AIS C, 3 AIS D; level of injury C3–T12; 9.1 ± 16.7 years post-injury) who completed the training, their exercise adherence was 99%; 10 participants completed 30 out of 30 sessions and 1 participant completed 28 out of 30 sessions. The participants reported an average of 64 RPM, ranging from 50 to 87. Commonly reported side effects were stiff or tight shoulders (n = 6) and/or a sore neck (n = 3) in week 1 and 2 but the symptoms did not persist. Most participants increased the resistance of the arm bike after 2 weeks into the intervention to maintain the exercise at moderate intensity.

### Corticospinal excitability

**Figure 4:**
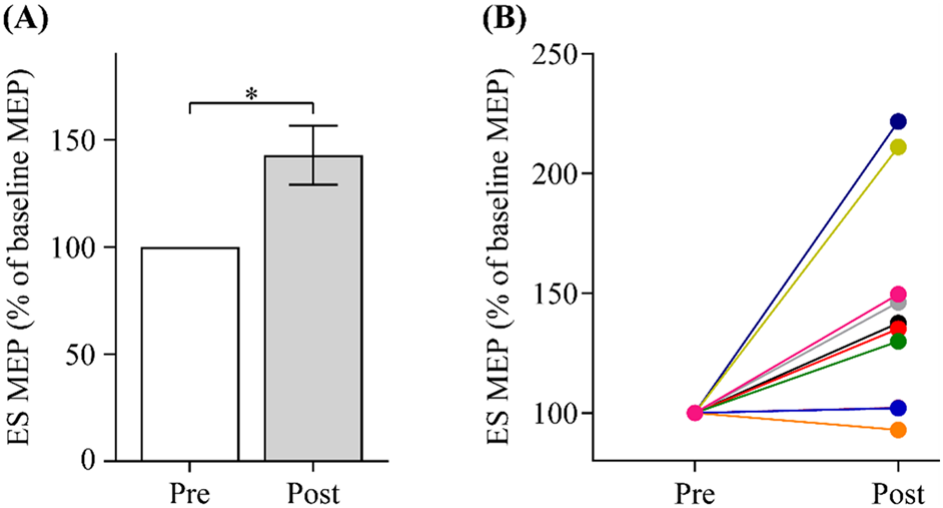
MEP data. (**A**) Group data (n = 10) showing increased MEP amplitudes of the ES after 6 weeks of the arm cycling exercise training in participants with SCI. Error bars represent the standard error of the mean. (**B**) The majority of the participants with SCI show greater ES MEPs at post-training. *p < 0.05.

Since all the participants enrolled in Experiment 2 took part in Experiment 1, the ES MEPs at pre-assessment were taken from the baseline MEPs obtained in Experiment 1. One participant (P14) did not have MEP data at post-assessment due to a technical issue with the TMS. Group results (n = 10) showed increased MEP amplitudes of the ES after 6 weeks of arm cycling exercise training in individuals with SCI (Z = − 2.497, *p* = 0.013; Fig. 4A). Note that most participants demonstrate greater ES MEP amplitudes post-assessment (Fig. 4B). There was no change in MEP latencies after the training (Z = − 1.48; *p* = 0.14). Pre-stimulus background EMG of the ES was the same at pre-and post-assessment (Z = − 1.48; *p* = 0.14).

### Functional outcomes

One participant was unable to perform the tasks without support to the trunk and hence did not have kinematic or HDEMG data. Of 10 participants who completed the tasks, kinematic data were unable to be analysed due to missing markers in one participant during the rapid shoulder flexion task and from another participant during the external perturbation task.

**Figure 5:**
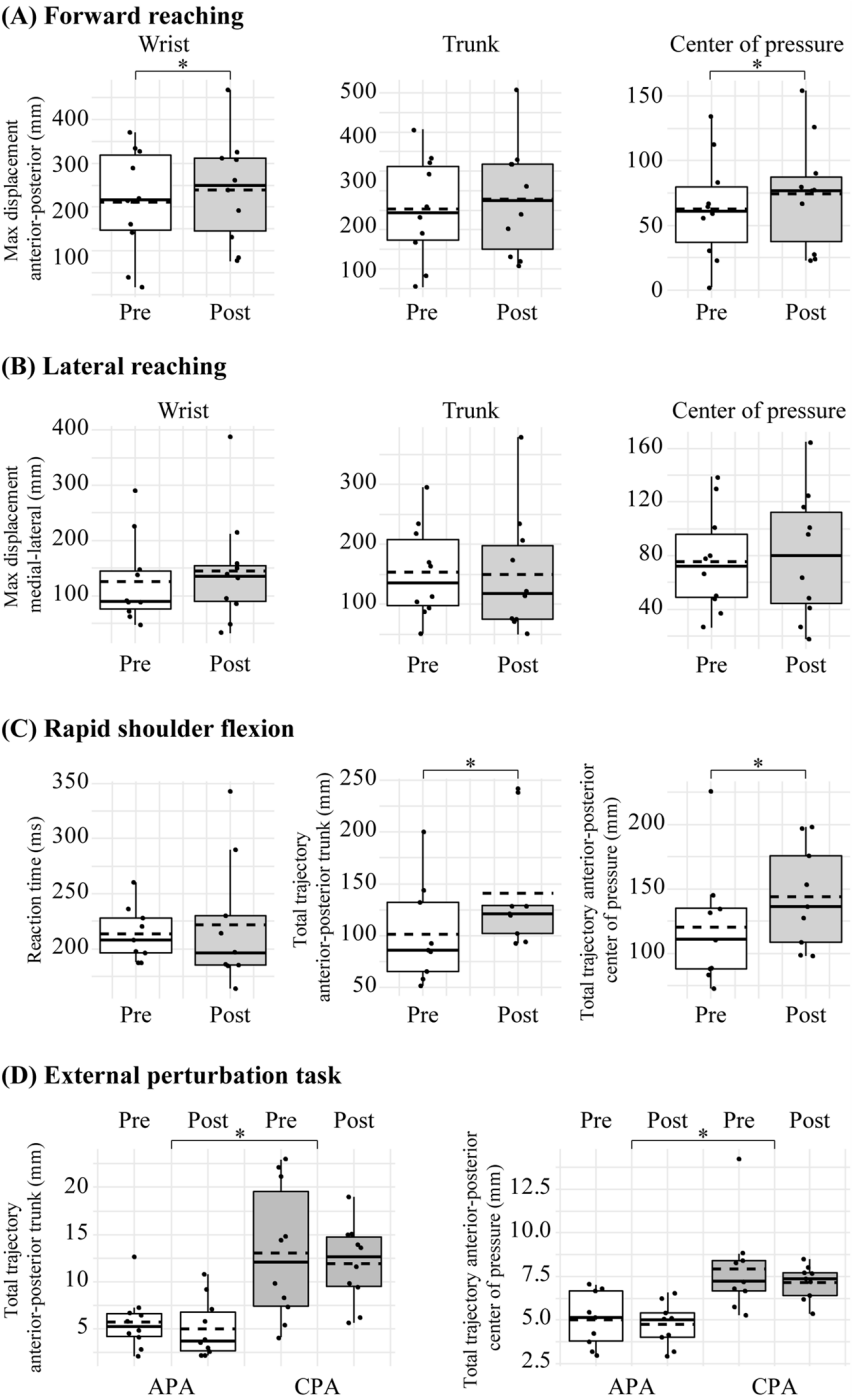
Functional results. Motion data group results for (**A**) forward reaching (n = 10), (**B**) lateral reaching (n = 10), (**C**) rapid shoulder flexion (n = 9), and (**D**) external perturbation task (n = 9). Two analysis windows were used in the external perturbation task: anticipatory postural adjustment (APA) window and compensatory postural adjustment (CPA) window. The boxes extend from the first quartile to the third quartile. The thin vertical lines extend up to 1.5 times the interquartile range. Solid horizontal lines depict the median, and the dotted horizontal lines represent the mean. *p < 0.05.

We found that participants reached further (Pre: mean 211.83 mm, SD 121.86; Post: mean 239.55 mm, SD 122.33; *t*(9) = − 2.400, *p* = 0.04, 95% CI − 53.85 to − 1.60) and had a greater centre of pressure displacement in the anterior direction (Pre: mean 62.79 mm, SD 39.62; Post: mean 74.40 mm, SD 43.21; *t*(9) = − 4.233, *p* = 0.002, 95% CI − 17.82 to − 5.41) during forward reaching after the arm cycling exercise training (Fig. 5A). However, the changes in trunk displacement did not reach statistical significance (Pre: median 194.48 mm, range 404.48; Post: median 224.82 mm, range 450.51; Z = − 0.89; *p* = 0.37). No differences were found in lateral reaching (Fig. 5B; all *p* > 0.05). For the rapid shoulder flexion task, participants increased the trajectory of the trunk (Pre: median 85.51 mm, range 148.82; Post: median 124.67 mm, range 185.12; Z = − 2.310, *p* = 0.021) and the centre of pressure (Pre: median 110.85 mm, range 153.05; Post: median 132.02 mm, range 100.10; Z = − 2.073, *p* = 0.038) in the anterior–posterior direction after the training (Fig. 5C), albeit the reaction time (calculated from the wrist markers) did not change (Pre: median 208.00 ms, range 72.00; Post: median 191.27 ms, range 179.00; Z = − 0.059, *p* = 0.953). Furthermore, repeated measures ANOVAs revealed an effect of window (APA vs. CPA) in the total trajectory of the trunk (Pre APA: mean 5.73 mm, SD 2.93; Post APA: mean 5.03 mm, SD 3.08; Pre CPA: mean 13.01 mm, SD 7.09; Post CPA: mean 11.95 mm, SD 4.20; *F*_1,9_ = 15.626, *p* = 0.003; η^2^p = 0.635) and centre of pressure (Pre APA: mean 5.02 mm, SD 1.57; Post APA: mean 4.73 mm, SD 1.26; Pre CPA: mean 7.93 mm, SD 2.65; Post CPA: mean 7.15 mm, SD 0.99; *F*_1,8_ = 11.971, *p* = 0.009; η^2^p = 0.599) during the external perturbation task, with large effect sizes (Fig. 5D). However, there was no effect of time or an interaction of window x time in the kinematic data (all *p* > 0.05) during the task.

### Neuromuscular function

The participant’s HDEMG data with low signal-to-noise ratios were excluded from the analysis (forward and lateral reaching n = 2; rapid shoulder flexion n = 2; external perturbation n = 2). Some participants having unsuccessful data recordings detected during post-processing were also excluded from the analysis (forward reaching n = 2; lateral reaching n = 1; rapid shoulder flexion: n = 1; external perturbation n = 3).

**Figure 6:**
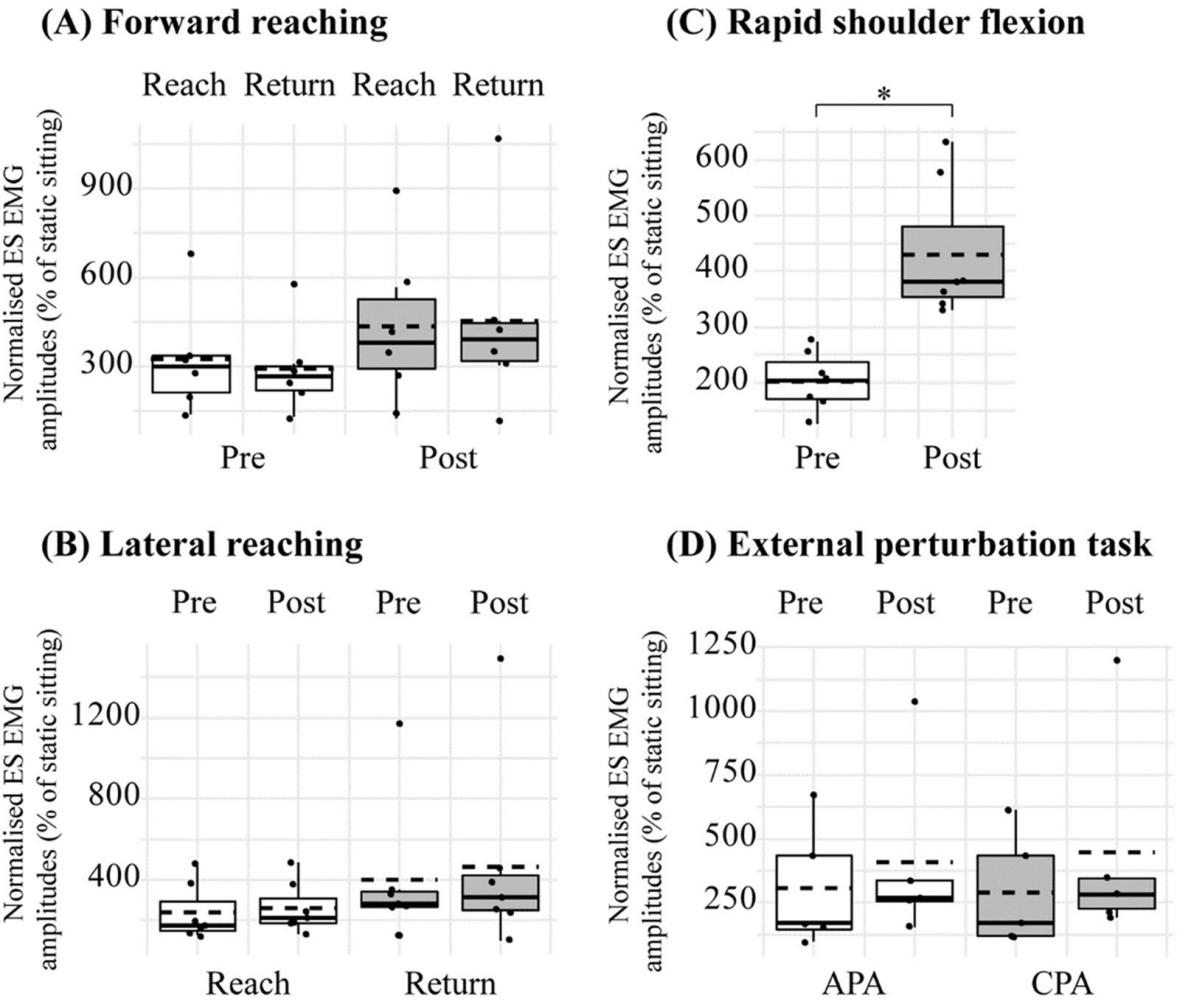
Normalised root-mean-square amplitudes of ES EMG in (**A**) forward reaching, (**B**) lateral reaching (**C**) rapid shoulder flexion, and (**D**) the external perturbation task. Note that the reaching tasks are separated into a reaching and returning phase. Two analysis windows were used in the external perturbation task: anticipatory postural adjustments (APA) and compensatory postural adjustments (CPA). The boxes extend from the first quartile to the third quartile. The thin vertical lines extend up to 1.5 times the interquartile range. Solid horizontal lines depict the median, and the dotted horizontal lines represent the mean. *p < 0.05.

Group results revealed increased normalised RMS amplitudes of the ES during the rapid shoulder flexion task at post-assessment (Pre: mean 202.67%, SD 52.16; Post: mean 430.12%, SD 122.29; *t*(6) = − 7.220, *p* < 0.001, 95% CI − 304.53 to − 150.36; Fig. 6C). However, there were no effects of phase, window, or time on normalised RMS amplitudes of the ES during forward reaching, lateral reaching or the external perturbation task (all *p* > 0.05; Fig. 6A,B,D).

## Discussion

Our findings show that ES MEPs increased after 30 min of arm cycling exercise in both individuals with SCI and healthy adults, suggesting exercise-induced acute neuroplasticity in the corticospinal pathways projecting to the ES. Moreover, we demonstrate that home-based, unsupervised arm cycling exercise training resulted in improvements in forward reaching distance, accompanied by a higher centre of pressure trajectory. The magnitude of the trunk movement as well as the global activity of the thoracic ES during the rapid shoulder flexion task was also increased after the training. These improvements were accompanied by increased corticospinal excitability of the ES, suggesting a change in neuromuscular control of the trunk underpinning the functional improvements after the training. Moreover, high adherence and no reports of any serious adverse events suggest the feasibility of the training program to be self-directed and performed unsupervised in the community.

Exercise is one of the common approaches for rehabilitation in humans with SCI^35,36^. Here we found that 6 weeks of home-based, unsupervised arm cycling exercise training (30 sessions in total), improved forward reaching distance in individuals with chronic incomplete SCI. This is in keeping with previous studies showing that a similar number of sessions of professional-led balance training or supervised kayak ergometers training^37,38^ improved dynamic sitting balance after SCI. Our participants with chronic incomplete SCI improved reaching distance by ~ 13%, which is comparable to what has been reported after 30 sessions of supervised, laboratory-based kayak ergometers training in people with SCI^37^. Additionally, our results are consistent with the effect of activity-based therapy in the community in a similar population^39,40^. Conversely, two previous studies reported no improvement in reaching distance after a similar exercise program involving the upper body in people with chronic SCI; one study delivered 15 sessions of group-based arm-crank exercise^17^, while the other study conducted 8 sessions of indoor wheelchair curling training^41^. This suggests the number of exercise sessions is an important consideration for trunk rehabilitation following SCI^42^. Note that prior work using supervised, task-specific balance exercise training reported improvements in lateral reaching distance in a similar population^43^, while we did not observe changes in lateral reaching after the intervention in our participants. Hence, the type^44^ (task-specific vs. upper-body vs. whole body exercise) and the setting^45^ (supervised vs. home-based) of exercise delivered may influence the magnitude of the effects of exercise for trunk rehabilitation following SCI. Moreover, increased forward reaching distance accompanied by increased trajectory of the centre of pressure, but not by increased trajectory of the upper trunk, may suggest the improvements in reaching distance was from increased movements of the lower back and pelvis^46^. Furthermore, we found that participants with SCI increased the magnitude of the trunk displacement and the centre of pressure trajectory in the opposite direction to the shoulder flexion, potentially better for the arms to move anteriorly, and this was accompanied by increased EMG amplitudes of the thoracic ES. This suggests improved recruitment of the ES and volitional control of the trunk during functional movements of the upper extremities after the arm cycling exercise training in individuals with SCI. Using surface bipolar EMG prior work has shown that unsupported upper-body exercise was effective in activating the trunk muscles^16,17^ that may explain the training-induced improvement in motor function seen in our study. The increased EMG activity of the trunk muscles after an intervention has been considered as a biomarker of improvement of motor function. One study reported improved voluntary activation of serratus anterior and lower trapezius in isolation after a targeted exercise intervention in adults with paraplegia^47^. Another study utilising combined transcutaneous electrical spinal cord stimulation and task-specific exercise for trunk function showed increased forward reaching distance, together with increased activity of a number of trunk muscles, including obliques, rectus abdominis, and latissimus dorsi^48^. However, while our participants improved their reaching distance, we did not observe increased EMG activity of the thoracic ES during forward reaching after the training. One explanation may be that our participants with SCI recruited other regions of the ES (e.g., lumbar ES) or other trunk and back muscles, as a result of improved motor function, to assist the arm moving to the front. Another explanation could be that the participant used more of the shoulder and scapular movements to assist the arm reaching forward after training. Although this is speculative, prior work showed active involvement of the shoulders and scapulae during arm cycling^49^. We suggest future studies measuring kinematics and muscle activity of other axial muscles in the upper body for better understanding of the benefits of arm cycling in activities of daily living. Moreover, the recordings were obtained on different days (pre- and post-assessment); this may introduce between-session variability to the EMG amplitudes. However, prior work using HDEMG showed excellent inter-session reliability of RMS amplitudes in thoracic ES in healthy adults^30^, and using HDEMG may be more robust than conventional EMG because of its larger recording area, allowing the possibility to record muscle activity from a larger portion of the muscle in contrast to the small recording area of bipolar EMG. More research in identifying changes in muscle synergies between regions of the trunk muscles using HDEMG in humans with SCI is needed.

Our results showed increased MEP amplitudes of the ES after 30 min of arm cycling exercise in both participants with and without SCI. This is consistent with prior work demonstrating increased corticospinal excitability of the ES after 30 min of unilateral arm cycling exercise in healthy adults^22^. This is also in agreement with evidence showing increased corticospinal excitability projecting to the ES during unilateral tonic contractions of elbow flexors in healthy adults^20^ and in people with chronic, incomplete SCI^21^. Importantly, we found increased MEP amplitudes of the ES after 6 weeks of arm cycling exercise training in participants with chronic SCI, suggesting training-induced plasticity in the corticospinal pathways projecting to the trunk muscles.

What are the mechanisms underlying increased corticospinal excitability of the trunk muscles after arm cycling exercise training? Previous studies have reported that neural interactions between the arms and trunk muscles is mediated, in part, in the primary motor cortex^20,22^. Following SCI, the corticospinal projections above and below the injured site undergo extensive reorganisation^50^. Animal studies have shown that propriospinal commissural interneurons can reconnect the injured corticospinal tract, forming new intraspinal circuits to receive descending commands from the corticospinal motor system, leading to improved motor function^51,52^. Additionally, activation of the corticospinal neurons projecting to the arm muscles during the arm cycling may interact with the corticospinal pathways to the trunk muscles via the propriospinal axons^53^ and the intraspinal branching in the dorsolateral column of the spinal cord^54,55^, inducing neuroplasticity and improvement of trunk control after training in our participants with SCI.

Research has shown increased activity, or co-activation, of trunk muscles during upper-body exercise in people with SCI^16,17^. It is possible that our results of increased MEP size resulted from direct activation of the trunk muscles during the arm cycling exercise. However, previous studies have shown changes^56^ or no change^57^ in corticospinal excitability of the trained muscle of the upper extremities after task-specific exercise training in individuals with SCI, possibly due to the contribution of other descending motor pathways^58,59^, in addition to the corticospinal tract, to the improvement of motor performance. Moreover, prior work has shown increased corticospinal output to the tibialis anterior after arm-leg cycling training, but not leg-only cycling training, in individuals with incomplete SCI^60^, indicating the contribution of dynamic arm movements to the strength of corticospinal connectivity between different parts of the body. Hence, we suggest both direct activation of the trunk muscles and indirect activation of the neural pathways projecting to the trunk muscles from the arms contributed to improved corticospinal output to the trunk muscles in our participants with SCI.

A limitation of our study is the age and sex differences between groups in Experiment 1, given that both factors could influence MEP amplitudes. Research suggests that the effect of age on MEP amplitudes varied between muscles^61–63^. For instance, input–output curves of MEPs were different in the first dorsal interosseous but the same in the vastus lateralis between young and older adults^62^. While the extent to which the effect of age on MEP amplitudes of the ES remains unclear, we normalised MEP amplitudes of the ES at post exercise to baseline, and compared the percentage of MEP amplitudes between groups, to control for the age effect on our results. Nevertheless, it is important for further studies to consider the effect of age and/or sex on training-induced neuroplasticity in the corticospinal pathways after SCI. Another limitation is the small sample size which could explain large confidence intervals of the functional outcomes. Caution should be taken when interpretating the results. Nevertheless, changes in the outcome measurements were consistent between participants and across different outcomes, underpinning the benefits of undertaking arm cycling exercise for trunk rehabilitation after SCI. Moreover, establishing the training effects of arm cycling on the trunk muscles in recumbent or supported sitting posture would have rehabilitation implications on those with severe injuries who are unable to sit upright without support. Given that current trunk rehabilitation requires supervision, our results posit a new option for persons living with SCI who wish to improve trunk control at home, that is low cost and may result in similar effects to other supervised trunk exercise, such as kayak ergometer exercise and whole-body exercise.

## Conclusions

We show that single session of arm cycling can induce acute neuroplasticity in the thoracic ES in individuals with a chronic incomplete SCI, and that 6 weeks of home-based arm cycling exercise training could improve forward reaching and volitional control of the trunk during movements of the upper extremities. These changes were accompanied by increases in activation of the thoracic ES and corticospinal output, suggesting that the training was able to engage the corticospinal pathway projecting to the trunk muscles. Our results highlight an alternative, low-cost method for trunk rehabilitation after SCI that may be undertaken by individuals themselves, outside a clinical setting and without supervision.

## Data Availability

The datasets from the current study are not publicly available because patient data need to be handled in accordance with the current data protection laws and ethical guidelines but are available from the corresponding author on reasonable request.
